# Bring on the brequinar: an approach to enforce the differentiation of myeloid-derived suppressor cells

**DOI:** 10.1172/JCI165506

**Published:** 2022-12-01

**Authors:** Natalie K. Horvat, Gregory B. Lesinski

**Affiliations:** Department of Hematology and Medical Oncology, Winship Cancer Institute of Emory University, Atlanta, Georgia, USA.

## Abstract

Myeloid-derived suppressor cells (MDSCs) hinder antitumor immunity in multiple cancer types. While brequinar (BRQ), an inhibitor of dihydroorotate dehydrogenase, shows cytotoxicity in hematological malignancy, it has not yet been adapted to attenuate MDSCs by augmenting bone marrow progenitors in breast cancer. In this issue of the *JCI*, Colligan et al. demonstrate that BRQ restored terminal differentiation of MDSCs. Using in vivo models of immunotherapy-resistant breast cancer, the authors uncovered a mechanism by which BRQ promoted myeloid cell differentiation by limiting their suppressive function and enhancing the efficacy of immune checkpoint blockade therapy. The findings offer insight into the biogenesis of MDSCs, provide an alternative avenue for cancers that remain unresponsive to conventional therapies, and may be extended to future translational studies in patients.

## Targeting MDSC in the bone marrow

Myeloid-derived suppressor cells (MDSCs) are a heterogeneous population of cells that regulate immune responses and limit the efficacy of cancer immunotherapies through a diverse collection of mechanisms (1). Among these mechanisms are the production of immune-suppressive cytokines, the secretion of enzymes that deplete key amino acids and direct immune engagement, and the elimination of tumor antigen–reactive lymphocytes. Concerted efforts to target MDSCs have focused on altering their trafficking to tumors via chemokine receptors (i.e., CCR2), their depletion (via 5‑fluorouracil), or the paralysis of specific mediators that directly suppress cytotoxic T cell function (such as arginase, ROS, or TGF-β) (2, 3). Although these approaches are founded on strong preclinical data, their widespread application across tumor types is likely limited for a number of reasons. First, molecular characterization of MDSCs has been particularly challenging, since these cells exist as a heterogeneous population, rather than as a subset of myeloid cells. Thus, further investigation is required to uncover specific profiles of MDSCs suitable for targeting. Second, it is likely that targeting of a single mediator is insufficient to overcome multiple redundant suppressive mechanisms that MDSC populations harbor. Third, these approaches are primarily geared toward circulating or tumor-resident MDSCs, rather than progenitors in the bone marrow that eventually give rise to the suppressive myeloid cells themselves. Conceptually, targeting bone marrow progenitors that differentiate into MDSCs in the setting of cancer offers a distinct advantage that may circumvent these issues. Given the role for MDSCs as a key barrier to antitumor immunity in multiple cancer types, effective pharmacologic agents targeting the genesis of these cells could shift the design of immunotherapy regimens.

## Versatility of brequinar

**Figure 1:**
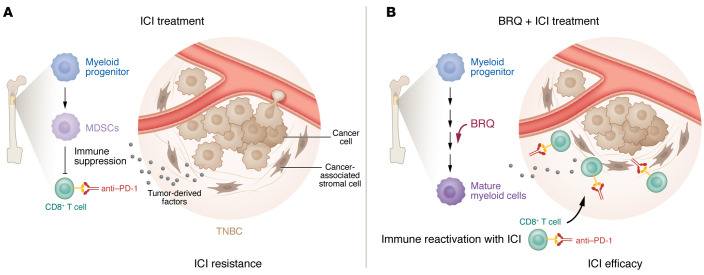
In therapy-resistant tumor models, BRQ reactivates the immune response to enhance the efficacy of immune checkpoint blockade therapy. (**A**) Because of the presence of MDSCs, TNBCs are resistant to ICIs, such as PD-1–targeted antibodies. MDSCs arise from bone marrow progenitors as a result of secreted tumor factors and act to suppress antitumor immunity. (**B**) Treatment with BRQ enforces myeloid biogenesis in the bone marrow, leading to myeloid progeny with limited immunosuppressive function. Combination treatment with BRQ and anti–PD-1 renders TNBCs responsive to treatment and leads to tumor regression.

In this issue of the *JCI*, Colligan et al. addressed the concept of targeting MDSCs at the bone marrow progenitor phase in a series of preclinical studies (4). The authors focused on immunotherapy-resistant breast cancer, since MDSCs play a prominent role in this tumor type, lending future translational importance to the study (5). The investigative team took a creative approach by adapting a dihydroorotate dehydrogenase (DHODH) inhibitor, brequinar (BRQ), as a means to accelerate myeloid cell maturation from progenitors in the bone marrow (Figure 1). This idea was inspired by prior observations from acute myeloid leukemia (AML) studies, in which DHODH inhibitors could terminally differentiate leukemic myeloid progenitors (6–8). From a mechanistic standpoint, the DHODH enzyme facilitates a rate-limiting step in pyrimidine synthesis, which ultimately enforces the terminal differentiation of myeloid cells. Importantly, the data on BRQ showed pyrimidine synthesis in MDSCs as an important mechanism that suppressed T cell function, since T cell proliferation was restored when MDSCs were treated with BRQ (6–8). This mechanism was then validated using leflunomide, another DHODH inhibitor, and the effects of BRQ were reversible by the addition of uridine, which allowed cultured cells to bypass the endogenous pathway of pyrimidine synthesis that required DHODH (4).

Using a series of both in vitro and in vivo studies, the authors showed the versatility of BRQ as a clever approach to interfere with MDSC biogenesis and subsequent functional activity. Rather than eliminating cells with an MDSC phenotype, this drug facilitated the differentiation of granulocyte-macrophage progenitors (GMPs) that led to the maturation and functional restoration of MDSCs. For example, in vivo administration of BRQ to mice with triple-negative breast cancer (TNBC) did not reduce splenic or peripherally circulating MDSC numbers, however it did affect their maturation, as evidenced by increases in CD101 (a neutrophil marker) and Ly6G and Ly6C on polymorphonuclear (PMN) MDSCs. Consequently, BRQ also reduced the ability of PMN-MDSCs to elicit a suppressive function on T cells and dampened the expression of multiple pathways and downstream functional mediators of MDSC biogenesis, including Arg1, iNOS, etc. (4). This feature is particularly attractive, as it suggests that BRQ targets both the differentiation and downstream functions of MDSCs in relevant models. Although BRQ lacked robust single-agent efficacy in tumor models, it did reduce the prevalence of spontaneous lung metastases and showed enhanced efficacy when combined with antibodies targeting the programmed cell death protein 1 (PD-1) and cytotoxic T-lymphocyte–associated antigen 4 (CTLA-4) immune checkpoints. Not surprisingly, these combinations elicited antitumor activity in a CD8^+^ T cell–dependent manner. Adding rigor to their findings that BRQ directly targets MDSC biogenesis were a series of adoptive transfer studies with IRF8-deficient MDSCs, which ultimately reversed the benefit of BRQ. Finally, the authors showed that inhibition of MDSC biogenesis by BRQ was generalizable in both the murine 4T1 tumor model and in a human bone marrow culture system. This report represents an innovative application of DHODH inhibitors as a means to augment the efficacy of immune checkpoint blockade in resistant tumor models (Figure 1) (4).

While the data point to modulation of endogenous pyrimidine synthesis as a culprit responsible for the antitumor action of BRQ, overlapping mechanisms probably also contribute to its effects on MDSC biogenesis. For example, clues arise from other strategies that enforce MDSC differentiation into macrophages and DCs, such as the use of al-*trans* retinoic acid (ATRA). This pharmacologic agent promotes myeloid differentiation through ERK1/2 pathway signaling and increased glutathione generation (9). The data in Colligan et al. implicate the dampening of oxidative stress as a potential mechanism of myeloid differentiation. In this study, BRQ-treated MDSCs showed a decrease in the unfolded protein response pathway and downregulation of inducible NOS (iNOS) enzymes, both of which are mechanisms known to increase ROS. These observations provide evidence that reducing oxidative species may also facilitate myeloid differentiation (4). In another recent report, other mechanisms of BRQ have emerged that elicit direct action on malignant cells, including its ability to increase lipid peroxidation in the inner mitochondrial membrane (10). For myeloid biogenesis, these studies collectively suggest that distinct oxidative species may catalyze specific programming of MDSC maturation. Based on these findings (10), BRQ may have some activity directly on tumor cells, with the caveat that single-agent BRQ did not result in robust growth inhibition in the breast cancer models used in the study by Colligan et al. (4).

## Conclusion

This enticing collection of data on BRQ encourages further investigation of its mechanism of action on MDSC biogenesis. For instance, it is tempting to speculate that BRQ might also act in part by modulating other critical regulators of MDSC biology such as IRF8 or other transcription factors that may align with gene expression data (11). Since transfer of IRF8-deficient MDSCs could retain the dominant suppressive features of these aggressive tumor models, even in the presence of BRQ, the impact of BRQ on IRF8 modulation deserves further study.

The data presented in Colligan et al. (4) support the need for continued investigation of BRQ and other DHODH inhibitors as a viable approach to counteract the predominance of suppressive myeloid cells in tumors, opening the door for more effective immunotherapies. Since BRQ is orally bioavailable with an established safety profile, it could be easily combined with immunotherapy as a means to alter MDSC biogenesis. Although this study focused on preclinical application of BRQ in combination with immune checkpoint inhibitors (ICIs) for breast cancer, it could be adapted to other tumor types with high levels of MDSCs in combination with other targeted or cellular immunotherapy approaches.

## Version 1 12/01/2022

Electronic publication
